# Association between cumulative lifetime exposure to reproductive hormones and tumor‐infiltrating lymphocytes, CD4 +, and CD8 + T cells among women with breast cancer

**DOI:** 10.1007/s00262-026-04471-3

**Published:** 2026-07-10

**Authors:** Yidan Zhang, Rand T. Akasheh, Angela R. Omilian, Bo Qin, Nur Zeinomar, Song Yao, Chi-Chen Hong, Elisa V. Bandera, Christine B. Ambrosone, Thaer Khoury, Ting-Yuan David Cheng

**Affiliations:** 1 Division of Cancer Prevention and Control, Department of Internal Medicine, The Ohio State University, 3650 Olentangy River Rd., Columbus, OH 43214 USA; 2 Ohio State University Comprehensive Cancer Center, The Ohio State University, Columbus, OH USA; 3 Department of Cancer Prevention and Control, Roswell Park Comprehensive Cancer Center, Buffalo, NY USA; 4 Cancer Epidemiology and Health Outcomes, Rutgers Cancer Institute, New Brunswick, NJ USA; 5 Department of Pathology & Laboratory Medicine, Roswell Park Comprehensive Cancer Center, Buffalo, NY USA; 6 Pelotonia Institute for Immuno-Oncology, The Ohio State University Comprehensive Cancer Center – James Cancer Hospital and Solove Research Institute, The Ohio State University, Columbus, United States

**Keywords:** Breast cancer, Tumor-infiltrating lymphocyte, Reproductive history, Hormone use, CD8-positive T-lymphocytes, CD4-positive T-lymphocytes

## Abstract

**Background:**

Reproductive hormones may influence immune responses to tumors. Tumor-infiltrating lymphocytes (TILs), CD4 +, and CD8 + T cell densities are markers of immune activity and breast cancer prognosis. We examined associations between lifetime reproductive hormone exposure, tumor immune infiltration, and survival among women with breast cancer.

**Methods:**

The study included invasive breast cancer cases in the Women's Circle of Health Study and the Women’s Circle of Health Follow-up Study. Lifetime endogenous hormone exposure (LHEendo) was defined as reproductive years plus pregnancy duration minus breastfeeding duration; lifetime exogenous hormone exposure (LHEexo) was defined as the combined cumulative duration of oral contraceptive and hormone replacement therapy use. TILs were scored as ordered percentage values from 0 to 100% stromal area; CD4 + /CD8 + T cell densities were quantified by immunohistochemistry. TILs were modeled using ordinal logistic regression, and CD4 + /CD8 + T cell densities were modeled using gamma generalized linear models among participants with positive density values, with covariates selected by directed acyclic graphs. Overall survival was assessed using Cox proportional hazards models, and breast cancer-specific survival was assessed using Fine-Gray competing-risk models.

**Results:**

Among 1195 women with valid TILs measurements, 553 and 610 had CD4 + and CD8 + T cell density data, respectively. Across all tumor molecular subtypes, a 1-year increment of LHEendo was associated with a 2.85% decrease in CD8 + T cell density (95% CI − 5.14%, − 0.61%). LHEendo was associated with lower odds of being in a higher TIL level among women with hormone receptor (HR) + /HER2- tumors (OR = 0.977, 95% CI 0.957, 0.997), although the interaction by molecular subtype was not statistically significant. LHEexo was associated with lower risk of all-cause mortality (HR = 0.979, 95% CI 0.960, 0.998). TIL-stratified analyses suggested that this association may be more apparent among women with TILs ≥ 50% (HR = 0.90, 95% CI = 0.82, 0.99; P-interaction = 0.10).

**Conclusions:**

Lifetime endogenous hormone exposure was associated with lower CD8 + T cell density overall, with exploratory findings of lower TIL levels among women with HR + /HER2- tumors. Lifetime exogenous hormone exposure was associated with a lower risk of all-cause mortality, with possible variation by TIL levels.

**Supplementary Information:**

The online version contains supplementary material available at https://doi.org/10.1007/s00262-026-04471-3.

## Background

Breast cancer remains a leading cause of cancer-related morbidity and mortality among women worldwide [1]. Reproductive hormones such as estrogen and progesterone drive breast tumor initiation and progression by modulating cell proliferation, apoptosis, and angiogenesis [2, 3]. Sex differences have been reported in patients’ responses to various anticancer immunotherapies. Specifically, men have demonstrated greater survival benefit from immune checkpoint inhibitors (ICIs) [4–6]. Such observations suggest that reproductive hormones may influence systemic immunity, and more importantly, reshape the tumor microenvironment (TME) by influencing lymphocytic infiltration, CD4 + /CD8 + T cell function, and the expression of cytokines in hormonally driven cancers like breast cancer.

Tumor-infiltrating lymphocytes (TILs) are mononuclear immune cells, predominantly T cells and B cells, that migrate into the tumor stroma or intratumoral areas, collectively constitute a key immune-cell component of the tumor microenvironment (TME) [7–9]. TILs have emerged as important indicators of the host immune response, with higher densities associated with improved outcomes, particularly in human epidermal growth factor receptor 2 (HER2)-positive and triple-negative subtypes [8–11]. CD4 + and CD8 + T cells are the two major T cell subsets in TILs, and both play a crucial role in recognizing and responding to tumor-associated antigens [9]. Measuring both overall TILs and these major T cell subsets provides a multidimensional view of the antitumor immune response. Previous studies showed that the level of immune-cell infiltrates is lower in estrogen receptor-positive (ER +) than ER-negative breast cancers [12, 13], suggesting a role of reproductive hormones in breast cancer immunity. Given the established prognostic relevance of TILs and CD8 + T cells in breast cancer, understanding how lifetime reproductive hormone exposure relates to these immune features may also help inform downstream survival outcomes. To date, there is no report on reproductive hormone exposure in association with overall TILs and the CD4 + and CD8 + T cell subsets.

This study aims to examine the associations of cumulative lifetime reproductive hormone exposures with overall TILs and with CD4 + and CD8 + T cell subsets in breast tumor tissue, and to evaluate their associations with overall and breast cancer-specific survival. We created separate measures for endogenous and exogenous hormone exposure to distinguish the effects of internally produced hormones driven by reproductive history from those of externally administered hormones through contraceptive or hormone therapy, which may differentially influence tumor immune infiltration and clinical outcomes. Because research using individuals with cancer have often selected covariates without a clear causal framework, risking both confounding and collider bias [14, 15], we used directed acyclic graphs (DAGs) to guide covariate selection, enabling robust control for confounding and minimizing collider bias.

## Methods

### Study population

In this study, we used breast cancer cases from the Women's Circle of Health Study (WCHS) and the Women’s Circle of Health Follow-up Study (WCHFS). The WCHS was a multi-site case–control study recruiting African American/Black and White women aged 20–75 years with newly diagnosed breast cancer in the New York metropolitan area and New Jersey from 2001 to 2013. Following the WCHS, the WCHFS enrolled Black women who lived in New Jersey with breast cancer diagnosed from 2013 to 2019, with ongoing follow-up. Details on recruitment procedures for the WCHS and WCHFS have been described elsewhere [16, 17].

All enrolled cases had no prior history of cancer, except for non-melanoma skin cancer. Upon obtaining consent, in-depth at-home interviews were conducted to collect questionnaire data on potential risk factors for breast cancer, including age, race, family health history, reproductive history, medical history, and lifestyle factors. Formalin-fixed paraffin-embedded (FFPE) tumor blocks and pathology reports from patients who provided consent for pathology and tissue release have been obtained from their hospitals. Pathology reports were reviewed, and hematoxylin and eosin (H&E) slides from FFPE blocks were prepared and examined by a board-certified breast pathologist (T.K.). Clinical and tumor characteristics, including the tumor grade, the American Joint Committee on Cancer (AJCC) stage, estrogen receptor (ER), progesterone receptor (PR), and human epidermal growth factor receptor 2 (HER2) were obtained from the pathology reports. Patients’ molecular subtypes were defined based on their ER, PR and HER2 status as hormone receptor-positive (HR + , i.e., ER + and/or PR +)/HER2 + , HR + /HER2-, HR-/HER2 + , and HR-/HER2-. None of the included patients received neoadjuvant therapy.

All study participants provided consent for the use of their data and specimens for research. The protocol was approved by all relevant Institutional Review Boards and conducted in accordance with the Declaration of Helsinki.

### Exposure

In the interview, data on reproductive risk factors were collected, including age at menarche, defined as the age of first menstrual period, and parity, defined as the number of pregnancies and their outcomes. For participants who had live births, additional questions were asked to determine the duration of each pregnancy in weeks or months, the month and year each pregnancy ended, whether they breastfed for each pregnancy, and the duration of each breastfeeding in months. Participants were asked for their menopausal status during the interview and were classified as postmenopausal if they reported having naturally stopped menstruation for at least one year before their date of diagnosis or had undergone bilateral oophorectomy. Participants were asked in the interview whether they had ever used oral contraceptives (OCs) or estrogen, progestins, or other female hormones for hormone replacement therapy (HRT), as well as the duration of their use.

Lifetime endogenous hormone exposure (LHEendo) was defined as the sum of reproductive years and cumulative pregnancy duration, minus cumulative breastfeeding duration. This measure was intended to provide a composite, duration-based summary of cumulative lifetime endogenous reproductive hormone exposure. Reproductive years were defined as the duration between age at menarche and age at diagnosis for premenopausal women, and as the duration between age at menarche and age at menopause for postmenopausal women. Cumulative pregnancy duration was defined as the number of pregnancies times 0.75, where 0.75 represents the average duration of each pregnancy in years; this approximation was used because complete duration information for each pregnancy was not consistently available across all participants. The cumulative pregnancy duration was treated as 0 for those who were nulliparous. The breastfeeding duration for individuals without breastfeeding was treated as 0.

Lifetime exogenous hormone exposure (LHEexo) was estimated by summing the years of OC or HRT use. Individuals who did not use HRT or OC were assigned an LHEexo value of 0.

### Outcome

#### Tumor-infiltrating lymphocytes (TILs) assessment

TIL levels were measured in the breast cancer tumor stroma using hematoxylin and eosin (H&E) stained sections from the formalin fixed, paraffin-embedded (FFPE) tissue blocks by the study pathologist based on International Immuno-oncology Biomarkers Working Group guidelines [8]. Briefly, the invasive tumor was selected and only TILs inside the tumor borders were evaluated. The stromal area within the invasive tumor was identified, excluding areas occupied by carcinoma cells. Stromal TILs were scored as a percentage of the stromal area occupied by lymphocytes, ranging from 0 to 100%. TIL scores were recorded as 0%, 1%, 10%, 20%, and subsequent 10% increments up to 100%. The distribution of stromal TILs was shown in Supplementary Fig. S1. The agreement in TILs scoring between the raters was acceptable (κ = 0.53–0.71) [18].

#### CD4 + and CD8 + T cell assessment

Tumor tissue microarrays (TMAs) were constructed from the FFPE tumor blocks. H&E-stained sections were reviewed by the study pathologist, with the outline of the tumor and embedded stroma manually circled. Three cores (0.6 mm in diameter) were randomly selected within the circled area. Although the punching is random within the circled area, the average across multiple cores is reasonably representative of the entire tumor with respect to the rate of stromal TILs (Spearman’s correlation coefficient = 0.56) [19]. CD4 and CD8 immunohistochemical (IHC) was performed by the Pathology Network Shared Resource (PNSR) at Roswell Park Comprehensive Cancer Center. IHC staining for CD4 was performed on TMAs using a mouse monoclonal anti-CD4 antibody (clone 4B12, Dako/Agilent, Santa Clara, CA, USA). CD8 staining was performed using a mouse monoclonal anti-CD8 antibody (clone C8/144B, Dako/Agilent, Santa Clara, CA, USA). The TMAs were sectioned at 4 μm. All TMAs were stained in a single batch using an autostainer. Detailed methods for staining are given elsewhere [11, 20].

Stained slides were digitally imaged at a magnification of 20X using the Aperio ScanScope XT (Leica Biosystems) digital slide scanner system. Aperio ImageScope was used for image visualization, annotation, and selection of regions of interest for analysis. Quantitative image analysis algorithm macros developed within the Aperio platform were used to assess IHC staining slides, generating outputs for the total number of analyzed cells, the percentage of CD4 + or CD8 + T cells, and the size of the analyzed tissue area. The CD4 + or CD8 + T cell density was calculated as the number of CD4 + or CD8 + T cells per mm^2^ of tumor tissue area to account for variability in tissue core sizes. TMA cores with insufficient tumor cellularity (< 20 tumor nuclei per core for CD4 and < 50 tumor nuclei per core for CD8) were excluded from the analysis. Only tumor cores were included in density calculations. The average CD4 + or CD8 + T cell density across multiple cores from each patient without weighting was used for statistical analyses. Personnel analyzing CD4, CD8, and TILs were masked from reproductive exposure and survival outcomes.

As an exploratory analysis to assess a functional immune-related feature, we evaluated the CD8 + T cell exhaustion signature previously developed by Yao et al. among participants with available NanoString gene-expression data [11].

#### Survival outcomes

Overall survival was defined as the time from breast cancer diagnosis to death from any cause. Breast cancer-specific survival was defined as the time from diagnosis to death due to breast cancer. Survival data were obtained from the WCHFS cohort. Because exposure data were collected after diagnosis, participants entered the survival risk set at enrollment rather than diagnosis to avoid immortal time bias. Vital status and cause of death were ascertained through active follow-up in WCHSF and linkage with cancer registry records among participants who lived in New Jersey. Participants were censored at the date of last contact or the end of follow-up (November 3, 2025), whichever occurred first.

### Covariate selection

We constructed separate DAGs for LHEendo and LHEexo in relation to immune markers and survival outcomes (Supplementary Figs. S2–S7). For LHEendo, DAGs were further stratified by menopausal status because the definition of LHEendo differed between premenopausal and postmenopausal women. Based on the DAGs, models assessing the association between LHEendo and tumor immune markers (overall TILs, CD4 + , and CD8 + T cell densities) and survival outcomes (overall survival and breast cancer-specific survival) were adjusted for race, family history of breast cancer (defined as self-reported breast cancer in a first-degree relative), and duration of OC use. For LHEexo, models for both tumor immune markers and survival outcomes were adjusted for race, age at diagnosis, menopausal status, and family history of breast cancer. AJCC stage and tumor grade were not included in the adjustment set because they were considered downstream variables that may be influenced by pre-diagnostic lifetime hormone exposure. BMI was not adjusted for because it was measured at enrollment after breast cancer diagnosis and therefore was not considered a confounder. For survival analyses, treatment variables were not included in the adjustment set because treatment occurs after diagnosis and is influenced by disease stage and tumor characteristics.

Age at diagnosis was not included in the LHEendo models. Among premenopausal women, LHEendo was defined as age at diagnosis minus age at menarche; therefore, additional adjustment for age at diagnosis resulted in multicollinearity, with variance inflation factors (VIFs) of 12.88, 13.40, 14.28, and 10.98 in the TILs, CD4 + T cell density, CD8 + T cell density, and overall survival models, respectively. Among postmenopausal women, LHEendo was defined as age at menopause minus age at menarche, and the exposure window was completed before diagnosis; therefore, age at diagnosis was not included in the DAG-defined adjustment set.

### Statistical analyses

Descriptive characteristics were summarized separately for individuals with TILs measurements available, those with both TILs and CD4 + T cell measurements, and those with both TILs and CD8 + T cell measurements. The associations of LHEendo and LHEexo with the tumor immune markers were evaluated separately for overall TILs, CD4 + T cell density, and CD8 + T cell density. Since stromal TILs were recorded as ordered percentage levels, ordinal logistic regression was used as the primary model to estimate associations of TIL level with LHEendo and LHEexo [21]. Odds ratios (ORs) were reported for the association of each 1-year increase in LHEendo or LHEexo with higher TIL level. The proportional odds assumption was evaluated using the Brant test, in which TIL values of 70%, 80%, 90%, and 100% were collapsed into a single 70–100% group because the upper TIL levels were sparse. In addition, we evaluated associations using fractional logit models as sensitivity analyses [22]. CD4 + and CD8 + T cell densities were analyzed as continuous measures. Both markers were non-negative, non-integer, and right skewed (Supplementary Fig. S1). The median CD4 + T cell density was 531.6 cells/mm^2^ (IQR: 247.5–1151.6; range 0.0–8787.9), and the median CD8 + T cell density was 71.5 cells/mm^2^ (IQR: 22.3–230.6; range: 0.0–5357.0). Zero-density observations were rare, occurring in 1 of 553 participants for CD4 + T cell density (0.18%) and 13 of 610 participants for CD8 + T cell density (2.13%). Therefore, associations with LHEendo and LHEexo were estimated using generalized linear models with a Gamma distribution and log link function among participants with positive CD4 + or CD8 + T cell density values. Regression coefficients (β) were converted to percentages using the formula: ${(e}^{\beta }-1)\times 100\%$, and interpreted as estimated percent change in expected mean CD4 + or CD8 + T cell density per 1-year increase in LHEendo or LHEexo. In addition, we performed sensitivity analyses using robust regression of log-transformed density among participants with positive density values. Linear regression models were used to estimate associations of LHEendo and LHEexo with the CD8 + T cell exhaustion signature, overall and stratified by race. To assess whether associations differed by type of LHEexo, OC duration and HRT duration were evaluated separately as sensitivity analyses for each outcome, using the same specifications and covariate adjustment as the primary analyses.

As a sensitivity analysis, these tumor immune markers were also converted into dichotomous variables to explore classifications that may help predict prognosis and guide treatment decisions [23]. Given that optimal thresholds for these biomarkers have not been established, the threshold for TILs was set at 50% (≥ 50% vs. < 50%), which has been shown previously to be associated with survival [11]. Thresholds for CD4 + and CD8 + T cell densities were defined using their respective cohort-specific medians (low vs. high expression) [20]. Logistic regression models were used to assess the associations of LHEendo and LHEexo with the dichotomous versions of overall TILs, as well as CD4 + and CD8 + T cell densities.

We conducted analyses of the association between LHEendo and LHEexo and those tumor immune markers, stratified by race (White and Black) and molecular subtypes. Molecular subtypes were categorized based on the HR and HER2 status: HR + /HER2 + ; HR + /HER2-; HR-/HER2 + ; and HR-/HER2-. The heterogeneity of associations in stratified analyses was assessed by including an interaction term for hormone exposure and the stratification variable in regression models. A likelihood ratio test was used to evaluate the significance of the interaction.

Association of LHEendo and LHEexo with overall survival were evaluated using Cox proportional hazards regression models, with hazard ratios (HRs) and 95% confidence intervals (CIs) estimated. Associations with breast cancer-specific mortality were evaluated using Fine-Gray competing-risk regression models implemented in the in the cmprsk package in R, with death due to causes other than breast cancer treated as competing events. Results from the competing-risk models were reported as subdistribution hazard ratios (SHRs) and 95% CIs. To assess potential effect modification by the tumor immune microenvironment, interaction terms between hormone exposure and tumor immune markers were included in the survival models. TILs were categorized as < 10%, 10–49%, and ≥ 50%. The proportional hazards assumption for Cox models was assessed using Schoenfeld residuals. For Fine-Gray models, proportionality of subdistribution hazards was evaluated using residual plots over event time.

Tests of statistical significance were two-sided. A *p* value of < 0.05 indicates significant results. Statistical analyses were performed using R version 4.4.2.

## Results

### Participant characteristics

Participants' characteristics are summarized in Table 1. A total of 1195 participants (858 Black and 337 White women) diagnosed with breast cancer and with TILs measurements were included in the study. Among them, 553 participants had data on CD4 + T cell density, and 610 had data on CD8 + T cell density. The analytic sample selection for the TILs, CD4 + , and CD8 + T cell analyses is shown in Supplementary Fig. S8. Among 1195 participants with TILs measurements, the mean lifetime exposure to endogenous and exogenous reproductive hormones was 35.7 and 5.20 years, respectively. The mean age at diagnosis was 53.0 years. The majority were postmenopausal (56.4%), with 61.9% having HR + /HER2- tumors. Nearly half (49.4%) were diagnosed at stage I, and 44.0% had high-grade tumors (Table 1). Most participants (81.0%) had no family history of breast cancer. A majority reported a history of oral contraceptive (OC) use (66.5%), while only 26.5% of postmenopausal women reported ever using hormone replacement therapy (HRT). Among those with additional CD4 + or CD8 + T cell data, the distributions of demographic, clinical, and exposure characteristics were generally similar. Participants in the CD4 + and CD8 + T cell subsets had higher proportions of Black individuals (80.1% and 77.4%, respectively) and higher percentages of AJCC stage II disease (44.7% and 44.1%, respectively) than those with TIL measurements overall (71.8% Black; 37.7% AJCC stage II).

**Table 1:** Descriptive characteristics of study participants

	Participants with TILs measurement N = 1195	Participants with both TILs and CD4 + T cell measurement N = 553	Participants with both TILs and CD8 + T cell measurement N = 610
Lifetime endogenous hormone exposure, years mean (SD)	35.7 (6.85)	36.1 (6.77)	36.0 (6.82)
Missing	14	11	11
Lifetime exogenous hormone exposure, years mean (SD)	5.20 (6.74)	5.34 (6.79)	5.39 (6.86)
Missing	19	11	12
Age at diagnosis, years mean (SD)	53.0 (10.5)	54.0 (10.4)	53.6 (10.5)
BMI at diagnosis, n (%)			
< 25	293 (24.6%)	118 (21.4%)	136 (22.3%)
≥ 25 and < 30	348 (29.2%)	148 (26.8%)	163 (26.8%)
≥ 30 and < 35	277 (23.2%)	148 (26.8%)	164 (26.9%)
≥ 35	275 (23.1%)	138 (25.0%)	146 (24.0%)
Missing	2	1	1
Race, n (%)			
Black	858 (71.8%)	443 (80.1%)	472 (77.4%)
White	337 (28.2%)	110 (19.9%)	138 (22.6%)
Family history of breast cancer, n (%)			
No	968 (81.0%)	448 (81.0%)	499 (81.8%)
Yes	227 (19.0%)	105 (19.0%)	111 (18.2%)
Menopausal status, n (%)			
Pre-menopause	521 (43.6%)	216 (39.1%)	246 (40.3%)
Post-menopause	674 (56.4%)	337 (60.9%)	364 (59.7%)
Molecular subtype, n (%)			
HR + /HER2 +	146 (12.9%)	64 (11.9%)	71 (11.9%)
HR + /HER2-	702 (61.9%)	342 (63.3%)	370 (62.1%)
HR-/HER2 +	70 (6.2%)	32 (5.9%)	35 (5.9%)
HR-/HER2-	217 (19.1%)	102 (18.9%)	120 (20.1%)
Missing	60	13	14
AJCC stage, n (%)			
I	557 (49.4%)	234 (42.5%)	261 (43.1%)
II	425 (37.7%)	246 (44.7%)	267 (44.1%)
III,IV	146 (12.9%)	70 (12.7%)	78 (12.9%)
Missing	67	3	4
Tumor grade, n (%)			
Low	177 (15.5%)	84 (15.5%)	94 (15.8%)
Intermediate	461 (40.5%)	199 (36.8%)	217 (36.5%)
High	501 (44.0%)	258 (47.7%)	284 (47.7%)
Missing	56	12	15
History of HRT use^1^, n (%)			
No	492 (73.5%)	252 (75.4%)	271 (75.1%)
Yes	177 (26.5%)	82 (24.6%)	90 (24.9%)
Missing	5	3	3
History of OC use, n (%)			
No	400 (33.5%)	171 (30.9%)	193 (31.6%)
Yes	795 (66.5%)	382 (69.1%)	417 (68.4%)

^1^Among postmenopausal women

HRT; hormone replacement therapy, OC; oral contraceptive, SD; standard deviation, TILs; Tumor-infiltrating lymphocytes

### Overall associations

Overall associations between lifetime hormone exposure and tumor immune markers are shown in Table 2. After adjustment for race, family history of breast cancer, and duration of OC use, LHEendo was not significantly associated with overall TIL levels. Each one-year increase in LHEendo was associated with lower odds of being in a higher TIL level, with a borderline significance (OR = 0.99; 95% CI 0.97, 1.00; P = 0.06). LHEexo was not associated with TIL level after adjustment for race, age at diagnosis, menopausal status, and family history of breast cancer (OR = 1.00; 95% CI 0.99, 1.02, P = 0.83). Sensitivity analyses collapsing sparse upper TIL values of ≥ 70% into a single group showed similar results and did not suggest violation of the proportional odds assumption. Results were similar in sensitivity analyses using fractional logit models (Supplementary Table S1).

**Table 2:** Association of hormone exposure with TILs, CD4⁺ T cells, and CD8⁺ T cells

Hormone exposure	Tumor immune markers	N	OR or percent change	95% CI	*p* value
LHEendo^1^	TIL level^3^	1169	OR = 0.99	(0.97, 1.00)	0.06
	CD4 + T cell density^4^	533	− 1.09%	(− 2.65, 0.45)	0.17
	CD8 + T cell density^4^	577	− 2.85%	(− 5.14, − 0.61)	**0.010**
LHEexo^2^	TIL level^3^	1176	OR = 1.00	(0.99, 1.02)	0.83
	CD4 + T cell density^4^	541	− 0.12%	(− 1.55, 1.42)	0.88
	CD8 + T cell density^4^	585	0.41%	(− 1.64, 2.65)	0.72

^1^Models using LHEendo as the predictor were adjusted for race, family history of breast cancer and the duration of oral contraceptive (OC) use

^2^Models using LHEexo as the predictor were adjusted for age at diagnosis, menopausal status, race, and family history of breast cancer

^3^TIL level were modeled using ordinal logistic regression and reported as odds ratio (ORs) for being in a higher TIL category per 1-year increase in hormone exposure. Sensitivity analyses using collapsed upper TIL categories showed similar results and did not suggest violation of the proportional odds assumption

^4^CD4 + and CD8 + T cell densities were treated as continuous variables. Generalized linear models with a Gamma distribution and log link function were used to assess the associations between hormone exposures and CD4 + or CD8 + T cell densities among participants with positive density values. Results are reported as percent changes in expected mean density per 1-year increase in hormone exposure

CI; confidence interval, LHEendo; lifetime endogenous hormone exposure, LHEexo; lifetime exogenous hormone exposure, TIL; Tumor-infiltrating lymphocyte

LHEendo was significantly associated with CD8 + T cell density in the primary gamma model restricted to participants with positive density values. Each one-year increase in LHEendo was associated with a 2.85% decrease in CD8 + T cell density (95% CI − 5.14%,− 0.61%; P = 0.010). LHEexo was not significantly associated with CD8 + T cell density (percent change per year: 0.41%; 95% CI − 1.64%, 2.65%; P = 0.72). No statistically significant associations were observed between either LHEendo or LHEexo and CD4 + T cell density. Sensitivity analyses using robust regression of log-transformed density showed similar directions of association but attenuated estimates, particularly for LHEendo and CD8 + T cell density (percent change per year: − 1.84%; approximate 95% CI − 3.87%, 0.24%; approximate P = 0.08).

### Stratified analyses

Stratified analyses of LHEendo by race and molecular subtype are presented in Supplementary Table S10. When stratified by race, LHEendo was not associated with TIL level among either Black women or White women, and there was no evidence of heterogeneity by race (P for interaction = 0.54). When stratified by molecular subtype, an inverse association between LHEendo and TIL level was observed among women with HR + /HER2- tumors (OR = 0.982; 95% CI 0.957, 0.997; P = 0.022). No associations were observed in other subtypes. However, the interaction test by molecular subtype was not statistically significant (P for interaction = 0.23); therefore, this finding was considered exploratory. Similar patterns were observed in fractional logit sensitivity analyses of TIL level stratified by race and tumor subtype (Supplementary Table S1).

For CD8 + T cell density, inverse associations with LHEendo were generally consistent across subgroups but did not reach statistical significance within individual race or molecular subtype strata, and no evidence of heterogeneity was observed. For CD4 + T cell density, no statistically significant associations with LHEendo were observed in any race or molecular subtype subgroup.

Stratified analyses of LHEexo and overall TILs as well as CD4 + and CD8 + T cell subsets are shown in Supplementary Table S11. LHEexo was not significantly associated with TIL levels, CD4 + T cell, or CD8 + T cell density in race- or subtype-specific analyses.

In an exploratory analysis among participants with available NanoString gene-expression data in 184 individuals (Supplementary Table S2), we evaluated the CD8 + T cell exhaustion signature previously developed by Yao et al. Neither LHEendo nor LHEexo was associated with the CD8 + T cell exhaustion signature overall or by race (Supplementary Table S3).

### Sensitivity analyses

When tumor immune markers were analyzed as dichotomous variables, results were generally consistent with the primary analyses using continuous measures. Overall, neither LHEendo nor LHEexo was associated with high TIL levels (≥ 50%) in the full study population (Supplementary Table S4). In analyses stratified by molecular subtype, LHEendo was inversely associated with high TIL level among women with HR + /HER2- tumors (OR per year: 0.94; 95% CI 0.90, 0.98; P = 0.006), with evidence of heterogeneity by subtype (P for interaction: 0.036, Supplementary Table S5). No significant associations between LHEendo and dichotomized CD4 + or CD8 + T cell densities were observed overall or across subgroups (Supplementary Tables S4 and S5).

For LHEexo, no significant associations with dichotomized TILs or CD4 + T cell density were observed overall (Supplementary Tables S4). In subtype-stratified analyses, LHEexo was positively associated with high CD8 + T cell density among women with HR-/HER2 + tumors (OR per year: 1.14; 95% CI 1.02, 1.28; P = 0.026), with evidence of heterogeneity by molecular subtype (*p* for interaction = 0.029, Supplementary Tables S6).

### Survival analysis

In the survival analytic dataset, the median follow-up time was 142.2 months (IQR: 112.0–189.9; range: 8.3–240.4 months). A total of 275 deaths occurred, including 144 breast cancer-specific deaths and 131 deaths from other causes; 668 participants were censored.

LHEexo was significantly associated with overall survival but not breast cancer-specific survival (Table 3). Each one-year increase in LHEexo was associated with lower risk of all-cause mortality (HR = 0.98; 95% CI, 0.96, 1.00; P = 0.029), with no evidence of violation of the proportional hazards assumption (Schoenfeld residual P = 0.13). In TIL-stratified analyses, each one-year increase in LHEexo was associated with a lower risk of all-cause mortality among women with TILs ≥ 50% (HR, 0.90; 95% CI, 0.82, 0.99, P = 0.027), whereas no associations were observed among women with TILs < 10% or 10–49% (P for interaction = 0.10). LHEexo was not associated with breast cancer-specific survival in the Fine-Gray model (SHR = 0.98; 95% CI, 0.95, 1.01; P = 0.11) overall or by TIL stratification. Visual assessment of residual plots did not suggest violation of the proportional subdistribution hazards assumption (Supplementary Fig. S9).

**Table 3:** Associations of exogenous hormone exposure^1^ with overall and breast cancer-specific survival by tumor-infiltrating lymphocyte categories

Outcome		Events/N	HR	95% CI	*p* value	P-interaction^4^
Overall survival^2^	All	272/926	0.98	(0.96, 1.00)	**0.029**	
	TILs < 10%	93/288	0.98	(0.95, 1.02)	0.29	0.10
	TILs 10–49%	155/526	0.99	(0.96, 1.01)	0.31	
	TILs ≥ 50%	24/112	0.90	(0.82, 0.99)	**0.027**	
Breast cancer-specific survival^3^	All	142/926	0.98	(0.95, 1.01)	0.11	
	TILs < 10%	42/288	0.94	(0.79, 1.12)	0.51	0.81
	TILs 10–49%	88/526	0.97	(0.94, 1.01)	0.16	
	TILs ≥ 50%	12/112	0.94	(0.79, 1.12)	0.51	

^1^Exposures were modeled as continuous variables per 1 year increase

^2^Cox models were adjusted for age at diagnosis, menopausal status, race, and family history of breast cancer

^3^Breast cancer-specific survival was analyzed using Fine-Gray competing-risk models, with deaths from non-breast cancer causes treated as competing events. HRs were reported as subdistribution hazard ratio (SHR)

^4^P-interaction values were from likelihood ratio χ^2^ test comparing models with vs. without the interaction term

HR; hazard ratio, CI; confidence interval, TILs, tumor-infiltrating lymphocytes

For LHEendo, however, the proportional hazard assumption was violated (Schoenfeld residual P = 0.0024; time-varying hazard ratios are reported in Supplementary Table S7). Thus, we reframe from testing the interaction and stratification by the immune markers.

In sensitivity analyses evaluating OC duration and HRT duration separately, results were generally consistent with the primary analyses and led to the same overall conclusion (Supplementary Table S8). Longer OC duration, but not HRT duration, was statistically significantly associated with a lower risk of all-cause mortality. In menopausal status-stratified sensitivity analyses, the inverse association between LHEendo and CD8 + T cell density observed in the overall analysis was more evident among premenopausal women, whereas no association was observed among postmenopausal women (Supplementary Table S9).

## Discussion

In this study, LHEendo was inversely associated with CD8 + T cell densities. Neither LHEendo nor LHEexo was significantly associated with overall TIL levels in the full study population. In stratified analyses by tumor subtype, LHEendo was inversely associated with overall TIL levels among women with HR + /HER2- tumors. Although the interaction test by molecular subtype was not statistically significant in the primary ordinal logistic model, sensitivity analyses using dichotomized TILs showed a similar inverse association in the HR + /HER2- subgroup and a statistically significant interaction by molecular subtype. Given the number of subgroup and sensitivity analyses conduced, this subtype-specific pattern should still be considered exploratory. LHEexo was not associated with CD4 + or CD8 + T cell density in the primary analyses, although exploratory analysis using dichotomized tumor immune markers suggested a positive association between LHEexo and CD8 + T cell densities among HR-/HER2 + tumors. In survival analysis, LHEexo was associated with lower risk of all-cause mortality. Exploratory TIL-stratified analyses suggested that this association may be more apparent among women with TILs ≥ 50%, although the interaction by TIL category was not statistically significant.

We observed an inverse association between LHEendo and CD8 + T cell densities among breast cancer patients in the primary gamma model, with the same direction of association observed in robust regression sensitivity analyses. This finding suggests that greater endogenous hormone exposure over a woman’s lifespan is associated with reduced cytotoxic T cell infiltration in the tumor microenvironment. Our results align with emerging animal and human studies suggesting that estrogenic signaling can modulate T cell function. Both in vitro and human studies have shown that antiestrogens, which reduce the effects of estrogen in the body, significantly increase CD8 + T cell infiltration in breast tumors [24, 25]. Several mechanisms, both dependent on and independent of the tumor’s estrogen receptor (ER) status, may underlie this negative association. In ER + tumors, estrogen modulates the immune response primarily by signaling through its two receptors, ERα and ERβ [26]. In a murine melanoma model, estrogen bound ERα to activate the ERα/estradiol signaling axis, which facilitated the differentiation and polarization of intratumoral macrophages toward an immunosuppressive phenotype, thereby indirectly reducing CD8 + T cell function and driving their exhaustion [27]. The same study also showed that estrogen modulation did not affect CD4 + T cell activation or proliferation, which was consistent with our findings of no association between LHEendo and CD4 + T cell density [27].

In addition, estrogen can act on ERα expressed by myeloid-derived suppressor cells (MDSCs), activating ERα signaling to phosphorylate STAT3 via upregulation of JAK2 in bone-marrow myeloid precursors. This drive MDSC mobilization into the tumor microenvironment, where they exert immunosuppressive functions and potentially enhance CD8 + T cell suppression across multiple tumor models, including breast cancer, regardless of the tumors’ ER status [28]. Estrogens and estrogen receptors can also remodel breast tumor glucose, amino acid, and lipid metabolism, creating an acidic, nutrient-depleted microenvironment that indirectly suppresses CD8 + T cell function [29, 30]. Taken together, given the central role of CD8 + T cell infiltration in antitumor immunity, our findings and prior animal and human studies raise the possibility that prolonged hormonal exposure may reduce the antitumor immune responses in breast cancer. The observation is also in line with epidemiological evidence that early menarche and no breastfeeding, factors that increase lifetime endogenous hormone exposure, are associated with increased risk of breast cancer in Black women [31–33].

We observed that higher LHEendo was associated with lower TIL levels in HR + /HER2- tumors, but not in other tumor subtypes. However, the interaction by tumor subtype was not statistically significant; therefore, this subtype-specific finding should be interpreted as exploratory. The underlying mechanism remains unclear, but one possible explanation is that endogenous hormone exposure may be related to lymphocyte trafficking in HR + /HER2- tumors through ER-associated regulation of chemokine signaling. Lymphocyte trafficking is a multistep process involving endothelial adhesion, chemokine-mediated activation, and migration within tissue. Chemokines are central to this process because they direct immune-cell recruitment and localization [34–36]. In particular, the CXCL12-CXCR4 axis is involved in T cell chemotaxis and migration [37], and CXCL12 has been identified as a target of estradiol in ER-positive breast cancer cells [38]. Estradiol has also been shown to regulate components of the CXCL12 signaling axis, including CXCL12, CXCR4, and CXCR7, in ER-positive breast cancer cells [39]. In addition, CCL2 and CCL5 have also been identified as estrogen-regulated chemokines, with higher extracellular levels observed in ER + cancers [40]. CCL2, also known as monocyte chemoattractant protein-1, has been shown to act as a T-lymphocyte chemoattractant [41]. These findings suggest that estrogen/ER-related signaling may alter the chemokines present in the tumor, which could influence lymphocyte recruitment or localization.

Alternatively, the inverse association between LHEendo and TILs in HR + /HER2- tumors may be mediated indirectly through non-lymphocyte components of the tumor microenvironment. For example, in estrogen-dependent breast cancer models, estradiol has been shown to increase CCL2 and CCL5 levels, promoting tumor-associated macrophage influx [40]. Because the polarization of tumor-associated macrophages, particularly M2-like macrophages, can contribute to immune suppression, this ER-mediated pathway may secondarily affect lymphocyte infiltration or activation within the tumor stroma [26, 27, 42]. In addition, the association between LHEendo and TILs in HR + /HER2- tumors may be mediated indirectly through fibroblasts. Prior evidence suggests that estrogen can activate stromal fibroblasts [43]. In breast cancer models, cancer-associated fibroblasts have been associated with an immunologically cold tumor microenvironment [44, 45]. Because TIL is a broader measure of lymphocytic infiltration and we did not directly assess chemokine expression, macrophage polarization, and fibroblast phenotypes, these mechanisms remain hypothesis-generating. Future studies incorporating detailed immunophenotyping are needed to answer whether overall or specific immune-cell components are influenced by LHEendo in HR + /HER2- tumors.

A statistically significant association was observed between LHEexo and CD8 + T cell densities in HR-/HER2 + tumors when the tumor immune marker level was dichotomized in the sensitivity analysis. When treated as continuous variables, a similar trend was observed, although it was not statistically significant. Our observations suggest that hormone exposure, even in tumors lacking classical hormone receptors, may potentiate antitumor immunity in HER2 + disease. Mechanistic studies are warranted to determine which exogenous hormone formulations effectively drive T cell recruitment and whether the evidence can be translated into improved responses to immunotherapy in HR-/HER2 + breast cancer.

We observed that LHEexo was associated with lower risk of all-cause mortality. In exploratory TIL-stratified analyses, the inverse association between LHEexo and all-cause mortality was more apparent among women with TILs ≥ 50%, whereas little association was observed among those with TILs < 10% or 10–49%; however, the interaction by TIL category was not statistically significant. A previous study using the same WCHS dataset reported that higher TIL level was associated with improved survival [11], supporting the prognostic relevance of tumor immune infiltration in this cohort. Our findings raise the possibility that the association between an upstream exposure such as exogenous hormone exposure and overall survival may vary according to the tumor’s baseline immune profile, with stronger associations observed in more immune-infiltrated tumors. More broadly, these findings raise the possibility that hormonal history and tumor immune status jointly affect survival, highlighting the importance of considering these factors together when studying breast cancer outcomes. These findings should, however, be interpreted in the context of prior literature showing that some menopausal hormone therapy regimens were associated with increased breast cancer incidence and mortality in initially cancer-free women in the Women’s Health Initiative (WHI) trial [46, 47]. Our LHEexo measure combined oral contraceptive and hormone replacement therapy use, making it not directly comparable to the specific hormone regimens evaluated in WHI. In sensitivity analyses separately evaluating OC and HRT duration, the association between combined LHEexo and overall survival appeared to be primarily attributed to OC duration. However, these component-specific analyses were exploratory. Future research is warranted on how OC and HRT influences tumor immune characteristics and clinical outcomes.

We used causal diagrams to guide covariate selection in our regression models to clarify potential sources of confounding and selection bias. In studies restricted to individuals with cancer, covariates adjustment without an explicit causal framework may introduce bias if variables affected by the exposure are adjusted for, or if the restriction to cancer cases opens noncausal paths between the exposure and outcome [14, 15]. For example, tumor stage or grade may lie downstream of pre-diagnostic hormone exposure; therefore, adjustment for these variables could block part of the paths through which hormone exposure may be related to tumor immune markers or survival. In addition, since our analyses were restricted to women diagnosed with breast cancer, breast cancer diagnosis may act as a collider if it is influenced by factors related to hormone exposure, such as OC use duration or family history of breast cancer, as well as other measured or unmeasured factors related to tumor immune markers or survival [14, 48]. Conditioning on breast cancer diagnosis may therefore open noncausal paths between hormone exposure and study outcomes [48–50]. To address this issue, we used DAG-specific adjustment sets to block measured noncausal paths between hormone exposure and study outcomes. However, since there may be unmeasured contributors to breast cancer diagnosis, residual collider bias cannot be ruled out.

This study has several strengths. First, we constructed composite variables that integrated age at menarche, age at menopause, pregnancy and breastfeeding history, and use of OC and HRT to quantify cumulative lifetime exposure to both endogenous and exogenous hormones. These measures reflect the overall hormonal environment across a woman’s reproductive lifespan, providing a more biologically meaningful estimate. Second, our large sample size, with a predominant representation of Black women, enhances the study’s generalizability. Third, we utilized centralized pathology review with standardized protocols for TIL scoring and quantification of CD4 + and CD8 + T cell densities using validated algorithms. Finally, we evaluated the tumor immune microenvironment by analyzing both overall stromal TIL levels and specific T cell subsets (CD4 + and CD8 + T cells). This dual-level analysis provides a more detailed view of immune infiltration by capturing both the broader lymphocytic infiltration in the tumor stroma as well as the functional composition of specific T cell populations. Nevertheless, the approach is not without limitations, primarily due to the sampling of TMA cores. In our pilot testing of CD8 + T cell density using IHC staining on TMA cores and whole sections taken from the same tumor blocks, the correlation was high (Pearson r = 0.83, p = 0.01). As we avoided the tumor margins when coring for TMA construction, our results do not apply to the tumor interface or other non-tumor regions.

Several limitations of our study should be noted. First, information on pre-diagnosis reproductive history and HRT/OC use was self-reported and collected after breast cancer diagnosis, which introduces the potential for recall errors. A prospective study with objective and longitudinal measures of sex hormones is warranted to confirm these findings. Second, the cumulative exogenous hormone exposure variable we defined was based solely on self-reported duration (in years) of OC and HRT use. Although we conducted sensitivity analyses evaluating OC and HRT duration separately, both the primary and sensitivity analyses were based on duration of use only and did not capture important details such as hormone type (estrogen-only vs. combined formulations), dosage, indication for use, recency, or changes in formulation over time. Therefore, we were unable to evaluate formulation-specific, or dose-specific effects on tumor immune infiltration or survival outcomes. Future studies should collect more detailed data on exogenous hormone type, formulation, dose, and indication to better characterize the potential effects of exogenous hormone exposure. Third, our LHEendo measure was based on the duration of reproductive hormone exposure and did not account for differences in exposure intensity across reproductive states. Specifically, pregnancy duration was estimated as the number of pregnancies multiplied by 0.75 years and was added to reproductive years without applying an additional intensity weight for the higher hormone concentrations during pregnancy. Because no established or validated weighting factor is available to translate pregnancy-related hormone intensity into an equivalent duration of reproductive years, future studies with more detailed reproductive hormone data may be able to better account for the intensity of pregnancy-related hormone exposure. Fourth, although we assessed stromal TILs and CD4 + and CD8 + T cell densities, these markers primarily reflect immune-cell abundance and do not fully capture functional immune states. We also evaluated a CD8 + T cell exhaustion signature in an exploratory NanoString subset; however, the small sample size and lack of additional functional immune markers, including regulatory T cells (Tregs), tumor-associated macrophages, and myeloid-derived suppressor cells, limit interpretation. Therefore, our findings should be viewed as hypothesis-generating and require confirmation in studies with more comprehensive immune profiling. Finally, as with all observational studies, we were unable to adjust for all potential confounders, and residual confounding may still be present. In particular, long-term OC and HRT users may differ from non-users in health awareness and social economic status, which were not fully measured in our data. These factors may influence both LHEexo and survival outcomes; therefore, findings related to LHEexo should be interpreted cautiously.

## Conclusions

In this observational study, longer lifetime endogenous hormone exposure was associated with lower CD8 + T cell density and showed an association with lower TIL levels in HR + /HER2- breast tumors, suggesting a potential relationship between cumulative endogenous hormone exposure and selected markers of the tumor immune microenvironment. The findings in HR + /HER2- should be considered as exploratory. Lifetime exogenous hormone exposure was associated with lower risk of all-cause mortality; exploratory analyses suggested that this association may be more apparent among women with higher TIL levels. Together, these findings suggest that endogenous and exogenous reproductive hormone exposures may have distinct relationships with tumor immune infiltration and clinical outcomes in breast cancer. Future studies incorporating detailed hormone formulation data and comprehensive functional and longitudinal immune profiling are warranted to clarify the mechanisms linking hormonal exposures, the tumor immune microenvironment, and survival.

## Supplementary Information

Below is the link to the electronic supplementary material.

Supplementary file1 (DOCX 1841 KB)

## Data Availability

The data supporting the findings of this study are not publicly available to protect patient privacy. The data will be made available to authorized researchers with the approval of the WCHS committee and relevant institutional review boards.
