# Supplementary material for: Association between cumulative lifetime exposure to reproductive hormones and tumor‐infiltrating lymphocytes, CD4 +, and CD8 + T cells among women with breast cancer
Source: Cancer Immunol Immunother. 2026 Jul 10;75(10):244. doi: 10.1007/s00262-026-04471-3 (PMC13638916; doi:10.1007/s00262-026-04471-3)
Supplement: Supplementary file 1 [file 262_2026_4471_MOESM1_ESM.docx]

**Supplementary Table S1. Sensitivity analysis of associations between lifetime hormone exposure and TIL level using fractional logit models**

| Hormone exposure | OR^1^ | 95% CI | P-value | P-interaction^8^ |
| --- | --- | --- | --- | --- |
| LHEendo |  |  |  |  |
| Overall^2^ | 0.99 | (0.98, 1.00) | 0.14 |  |
| Race^3^ |  |  |  | 0.37 |
| Black | 0.99 | (0.98, 1.01) | 0.36 |  |
| White | 0.98 | (0.96, 1.01) | 0.14 |  |
| Molecular subtype^4^ |  |  |  | 0.08 |
| HR+/HER2+ | 1.01 | (0.98, 1.04) | 0.38 |  |
| HR+/HER2- | 0.98 | (0.97, 1.00) | **0.014** |  |
| HR-/HER2+ | 1.02 | (0.99, 1.06) | 0.23 |  |
| HR-/HER2- | 0.99 | (0.97, 1.01) | 0.20 |  |
| LHEexo |  |  |  |  |
| Overall^5^ | 1.00 | (0.99, 1.01) | 0.59 |  |
| Race^6^ |  |  |  | 0.06 |
| Black | 1.01 | (1.00, 1.02) | 0.17 |  |
| White | 0.98 | (0.96, 1.01) | 0.18 |  |
| Molecular subtype^7^ |  |  |  | 0.46 |
| HR+/HER2+ | 0.98 | (0.94, 1.01) | 0.20 |  |
| HR+/HER2- | 1.00 | (0.99, 1.02) | 0.69 |  |
| HR-/HER2+ | 1.00 | (0.97, 1.03) | 0.83 |  |
| HR-/HER2- | 1.01 | (0.99, 1.03) | 0.34 |  |

1. ORs were reported per 1-year increase in hormone exposure.

2. Models were adjusted for race, family history of breast cancer and the duration of oral contraceptive (OC) use

3. Models were adjusted for race (included as an interaction term), family history of breast cancer and the duration of oral contraceptive (OC) use.

4. Models were adjusted for molecular subtype (included as an interaction term), race, family history of breast cancer and the duration of oral contraceptive (OC) use.

5. Models were adjusted for age at diagnosis, menopausal status, race, and family history of breast cancer.

6. Models were adjusted for race (included as an interaction term), age at diagnosis, menopausal status, and family history of breast cancer.

7. Models were adjusted for molecular subtype (included as an interaction term), age at diagnosis, menopausal status, race, and family history of breast cancer.

8. P-interaction values were obtained from Wald tests of the interaction term between hormone exposure and race or molecular subtype

**Supplementary Table S2. Descriptive characteristics of participants with available NanoString gene-expression data**

|  | Participants with Nanostring data  N=184 |
| --- | --- |
| Lifetime endogenous hormone exposure, years mean (SD) | 35.6 (6.85) |
| Missing | 5 |
| Lifetime exogenous hormone exposure, years mean (SD) | 5.33 (6.99) |
| Missing | 3 |
| Age at diagnosis, years mean (SD) | 53.2 (10.0) |
| BMI at diagnosis, n (%) |  |
| <25 | 40 (21.7%) |
| ≥25 and <30 | 51 (27.7%) |
| ≥30 and <35 | 47 (25.5%) |
| ≥35 | 46 (25.0%) |
| Race, n (%) |  |
| Black | 140 (76.1%) |
| White | 44 (23.9%) |
| Family history of breast cancer, n (%) |  |
| No | 149 (81.0%) |
| Yes | 35 (19.0%) |
| Menopausal status, n (%) |  |
| Pre-menopause | 81 (44.0%) |
| Post-menopause | 103 (56.0%) |
| Molecular subtype, n (%) |  |
| HR+/HER2+ | 29 (15.8%) |
| HR+/HER2- | 77 (41.8%) |
| HR-/HER2+ | 17 (9.2%) |
| HR-/HER2- | 61 (33.2%) |
| AJCC stage, n (%) |  |
| I | 85 (48.3%) |
| II | 73 (41.5%) |
| III,IV | 18 (10.2%) |
| Missing | 8 |
| Tumor grade, n (%) |  |
| Low | 19 (10.6%) |
| Intermediate | 54 (30.2%) |
| High | 106 (59.2%) |
| Missing | 5 |
| History of HRT use^1^, n (%) |  |
| No | 75 (74.3%) |
| Yes | 26 (25.7%) |
| Missing | 2 |
| History of OC use, n (%) |  |
| No | 64 (34.8%) |
| Yes | 120 (65.2%) |

^1^ Among postmenopausal women

HRT, hormone replacement therapy; OC, oral contraceptive; SD, standard deviation

**Supplementary Table S3. Association of hormone exposure with CD8+ T cell exhaustion signature^1^**

| Hormone exposure | N | β^2^ | 95% CI | P-value | P-interaction^5^ |
| --- | --- | --- | --- | --- | --- |
| LHEendo^3^ |  |  |  |  |  |
| Overall | 178 | 0.008 | (-0.002, 0.018) | 0.13 |  |
| Race - Black | 135 | 0.009 | (-0.002, 0.020) | 0.12 | 0.63 |
| Race - White | 43 | 0.003 | (-0.019, 0.025) | 0.79 |  |
| LHEexo^4^ |  |  |  |  |  |
| Overall | 181 | 0.006 | (-0.004, 0.016) | 0.23 |  |
| Race - Black | 137 | 0.006 | (-0.005, 0.017) | 0.27 | 0.96 |
| Race - White | 44 | 0.006 | (-0.018, 0.030) | 0.64 |  |

^1^ The CD8+ T cell exhaustion signature was previously developed by Yao et al.(1). Analyses were restricted to participants with available NanoString gene-expression data.

^2^ β coefficients and 95% CIs represent the estimated change in CD8+ T cell exhaustion signature score associated with a 1-year increase in LHEendo or LHEexo.

^3^ Models for LHEendo were adjusted for race, family history of breast cancer and the duration of oral contraceptive (OC) use.

^4^ Models for LHEexo were adjusted for age at diagnosis, menopausal status, race, and family history of breast cancer

^5^ P-interaction was calculated using a likelihood ratio χ2 test comparing models with and without the hormone exposure-by-race interaction term.

CI, confidence interval; LHEendo, lifetime endogenous hormone exposure; LHEexo, lifetime exogenous hormone exposure.

**Supplementary Table S4. Association of hormone exposure with TILs, CD4⁺ T cells, and CD8⁺ T cells (as dichotomous variables)**

| Hormone exposure | Tumor immune marker | OR | 95% CI | P-value |
| --- | --- | --- | --- | --- |
| LHEendo^1^ | TIL level^3^ | 0.98 | (0.96, 1.01) | 0.22 |
|  | CD4+ T cell density ^4^ | 0.99 | (0.96, 1.01) | 0.30 |
|  | CD8+ T cell density ^4^ | 0.98 | (0.96, 1.00) | 0.11 |
| LHEexo^2^ | TIL level^3^ | 1.01 | (0.98, 1.03) | 0.55 |
|  | CD4+ T cell density ^4^ | 0.99 | (0.96, 1.01) | 0.39 |
|  | CD8+ T cell density ^4^ | 1.01 | (0.98, 1.03) | 0.53 |

1. When fitting model using LHEendo as the predictor, models were adjusted for race, family history of breast cancer and the duration of oral contraceptive (OC) use

2. When fitting model using LHEexo as the predictor, models were adjusted for age at diagnosis, menopausal status, race, and family history of breast cancer.

3. TILs were treated as a dichotomous variable, using 50% as the cut-off (≥50% vs. <50%). Logistic regression models were used to assess the association between hormone exposure and TILs

4. CD4+ and CD8+ T cell densities were treated as dichotomous variables using their respective medians as cut-offs. Logistic regression models were used to assess the associations between hormone exposure and CD4+ or CD8+ T cell densities

OR, odds ratio; CI, confidence interval; LHEendo, lifetime endogenous hormone exposure; LHEexo, lifetime exogenous hormone exposure; TILs, Tumor-infiltrating lymphocytes

**Supplementary Table S5. Stratified analyses of endogenous hormone exposure in relation to TILs, CD4⁺ T cells, and CD8⁺ T cells by race and molecular subtype (as dichotomous variables)**

| Subgroup | OR | 95% CI | P-value | P-interaction^5^ |
| --- | --- | --- | --- | --- |
| *TIL level^3^* |  |  |  |  |
| Race^1^ |  |  |  | 0.36 |
| Black | 0.99 | (0.96, 1.02) | 0.40 |  |
| White | 0.95 | (0.88, 1.03) | 0.19 |  |
| Molecular subtype^2^ |  |  |  | **0.036** |
| HR+/HER2+ | 1.04 | (0.95, 1.13) | 0.40 |  |
| HR+/HER2- | 0.94 | (0.90, 0.98) | **0.006** |  |
| HR-/HER2+ | 1.06 | (0.97, 1.15) | 0.17 |  |
| HR-/HER2- | 0.98 | (0.94, 1.03) | 0.48 |  |
| *CD4+ T cell density^4^* |  |  |  |  |
| Race^1^ |  |  |  | 0.28 |
| Black | 0.99 | (0.96, 1.01) | 0.35 |  |
| White | 0.96 | (0.91, 1.01) | 0.10 |  |
| Molecular subtype^2^ |  |  |  | 0.36 |
| HR+/HER2+ | 1.04 | (0.97, 1.13) | 0.26 |  |
| HR+/HER2- | 0.98 | (0.94, 1.01) | 0.14 |  |
| HR-/HER2+ | 1.01 | (0.91, 1.11) | 0.87 |  |
| HR-/HER2- | 1.01 | (0.95, 1.07) | 0.76 |  |
| *CD8+ T cell density^4^* |  |  |  |  |
| Race^1^ |  |  |  | 0.28 |
| Black | 0.99 | (0.96, 1.01) | 0.35 |  |
| White | 0.96 | (0.91, 1.01) | 0.10 |  |
| Molecular subtype^2^ |  |  |  | 0.87 |
| HR+/HER2+ | 1.01 | (0.94, 1.09) | 0.73 |  |
| HR+/HER2- | 0.98 | (0.95, 1.01) | 0.28 |  |
| HR-/HER2+ | 0.98 | (0.89, 1.08) | 0.71 |  |
| HR-/HER2- | 0.98 | (0.92, 1.03) | 0.39 |  |

1. Models were adjusted for race (included as an interaction term), family history of breast cancer and the duration of oral contraceptive (OC) use.

2. Models were adjusted for molecular subtype (included as an interaction term), race, family history of breast cancer and the duration of oral contraceptive (OC) use.

3. TILs were treated as a dichotomous variable, using 50% as the cut-off (≥50% vs. <50%). Logistic regression models were used to assess the association between LHEendo and TILs.

4. CD4+ and CD8+ T cell densities were treated as dichotomous variables using their respective medians as cut-offs. Logistic regression models were used to assess the association between LHEendo and CD4+ or CD8+ T cell densities.

5. P-value of the likelihood ratio χ2 test for interaction.

OR, odds ratio; CI, confidence interval; LHEendo, lifetime endogenous hormone exposure; TILs, Tumor-infiltrating lymphocytes

**Supplementary Table S6. Stratified analyses of exogenous hormone exposure in relation to TILs, CD4⁺ T cells, and CD8⁺ T cells by race and molecular subtype (as dichotomous variables)**

| Subgroup | OR | 95% CI | P-value | P-interaction^5^ |
| --- | --- | --- | --- | --- |
| *TIL level^3^* |  |  |  |  |
| Race^1^ |  |  |  | **0.049** |
| Black | 1.02 | (0.99, 1.05) | 0.22 |  |
| White | 0.92 | (0.82, 1.03) | 0.14 |  |
| Molecular subtype^2^ |  |  |  | 0.30 |
| HR+/HER2+ | 0.92 | (0.81, 1.06) | 0.24 |  |
| HR+/HER2- | 1.02 | (0.97, 1.06) | 0.50 |  |
| HR-/HER2+ | 0.98 | (0.91, 1.05) | 0.59 |  |
| HR-/HER2- | 1.03 | (0.98, 1.08) | 0.20 |  |
| *CD4+ T cell density^4^* |  |  |  |  |
| Race^1^ |  |  |  | 0.86 |
| Black | 0.99 | (0.96, 1.02) | 0.49 |  |
| White | 0.98 | (0.93, 1.04) | 0.59 |  |
| Molecular subtype^2^ |  |  |  | 0.07 |
| HR+/HER2+ | 0.94 | (0.86, 1.02) | 0.15 |  |
| HR+/HER2- | 0.99 | (0.96, 1.02) | 0.44 |  |
| HR-/HER2+ | 1.10 | (0.99, 1.22) | 0.069 |  |
| HR-/HER2- | 0.97 | (0.91, 1.03) | 0.32 |  |
| *CD8+ T cell density^4^* |  |  |  |  |
| Race^1^ |  |  |  | 0.87 |
| Black | 1.01 | (0.98, 1.04) | 0.53 |  |
| White | 1.00 | (0.96, 1.05) | 0.87 |  |
| Molecular subtype^2^ |  |  |  | **0.029** |
| HR+/HER2+ | 0.94 | (0.87, 1.02) | 0.14 |  |
| HR+/HER2- | 1.00 | (0.97, 1.04) | 0.79 |  |
| HR-/HER2+ | 1.14 | (1.02, 1.28) | **0.026** |  |
| HR-/HER2- | 1.01 | (0.96, 1.07) | 0.67 |  |

1. Models were adjusted for race (included as an interaction term), age at diagnosis, menopausal status, and family history of breast cancer.

2. Models were adjusted for molecular subtype (included as an interaction term), age at diagnosis, menopausal status, race, and family history of breast cancer.

3. TILs were treated as a dichotomous variable, using 50% as the cut-off (≥50% vs. <50%). Logistic regression models were used to assess the association between LHEexo and TILs.

4. CD4+ and CD8+ T cell densities were treated as dichotomous variables using their respective medians as cut-offs. Logistic regression models were used to assess the association between LHEexo and CD4+ or CD8+ T cell densities.

5. P-value of the likelihood ratio χ2 test for interaction.

OR, odds ratio; CI, confidence interval; LHEexo, lifetime exogenous hormone exposure; TILs, Tumor-infiltrating lymphocytes.

**Supplementary Table S7. Time-varying hazard ratios for the association between endogenous hormone exposure and overall survival**

| Time month | HR^2^ | 95% CI |
| --- | --- | --- |
| 12 | 0.96 | (0.92, 1.01) |
| 24 | 0.98 | (0.95, 1.01) |
| 60 | 1.01 | (0.99, 1.03) |
| 120 | 1.03 | (1.00, 1.05) |
| 180 | 1.04 | (1.01, 1.07) |
| 240 | 1.05 | (1.01, 1.09) |

^1^ LHEendo were modeled as continuous variables per 1-year increase.

^2^ Time-varying hazard ratios were estimated for the primary LHEendo-overall survival Cox model because the proportional hazards assumption was not fully satisfied. Models were adjusted for race, family history of breast cancer and the duration of oral contraceptive (OC) use.

HR, hazard ratio; CI, confidence interval, LHEendo, lifetime endogenous hormone exposure.

**Supplementary Table S8. Sensitivity analyses evaluating hormone replacement therapy duration and oral contraceptive duration separately**

| Outcome | Exposure | N^6^ | Effect estimates^1^ | Estimate (95% CI) | P-value |
| --- | --- | --- | --- | --- | --- |
| TIL level | HRT duration | 1188 | OR^2^ | 1.01 (0.97, 1.05) | 0.69 |
|  | OC duration | 1183 |  | 1.00 (0.98, 1.02) | 0.98 |
| CD4+ T cell density | HRT duration | 549 | Percent change^3^ | 1.65 (-1.69, 5.79) | 0.39 |
|  | OC duration | 544 |  | -0.67 (-2.25, 1.04) | 0.44 |
| CD8+ T cell density | HRT duration | 594 | Percent change^3^ | 3.44 (-0.95, 9.43) | 0.21 |
|  | OC duration | 588 |  | -0.68 (-3.02, 1.87) | 0.58 |
| Overall survival | HRT duration | 274/937 | HR^4^ | 0.99 (0.95, 1.03) | 0.63 |
|  | OC duration | 273/932 |  | 0.97 (0.95, 1.00) | **0.021** |
| Breast cancer-specific survival | HRT duration | 144/937 | SHR^5^ | 1.01 (0.95, 1.07) | 0.82 |
|  | OC duration | 142/932 |  | 0.97 (0.94, 1.00) | 0.05 |

^1^ Effect estimates are presented per 1-year increase in hormone replacement therapy (HRT) duration or oral contraceptive (OC) duration.

^2^ Odds ratios (ORs) were estimated from ordinal logistic regression models for TILs category.

^3^ CD4+ and CD8+ T cell densities were treated as continuous variables. Generalized linear models with a Gamma distribution and log link function were used to assess the associations between HRT or OC duration and CD4+ and CD8+ T cell densities among participants with positive density values. Results are reported as percent changes in expected mean density per 1-year increase in HRT or OC duration.

^4^ Hazard ratios (HRs) were estimated from Cox proportional hazards models for overall survival.

^5^ Subdistribution hazard ratio (SHRs) were estimated from Fine-Gray competing risks models for breast cancer specific survival.

^6^ N for overall survival and Breast cancer-specific survival presented as the number of events/the number of total observations

^7^ All models used the same covariate adjustment as the corresponding primary analyses.

CI, confidence interval; TILs, tumor-infiltrating lymphocytes; HRT, hormone replacement therapy; OC, oral contraceptive.

**Supplementary Table S9. Menopause status-stratified associations between lifetime endogenous hormone exposure and TILs, CD4⁺ T cells, CD8⁺ T cells, and survival outcomes**

| Outcome | Menopausal status | N^7^ | Effect estimates^1^ | Estimate (95% CI)^4^ | P-value |
| --- | --- | --- | --- | --- | --- |
| TIL level | Premenopasual | 513 | OR^2^ | 0.99 (0.97, 1.01) | 0.43 |
|  | Postmenopasual | 656 |  | 1.01 (0.98, 1.04) | 0.55 |
| CD4+ T cell density | Premenopasual | 209 | Percent change^3^ | -1.91 (-4.27, 0.45) | 0.10 |
|  | Postmenopasual | 324 |  | 0.52 (-2.06, 2.98) | 0.68 |
| CD8+ T cell density | Premenopasual | 236 | Percent change^3^ | -4.03 (-7.25, -0.72) | **0.014** |
|  | Postmenopasual | 341 |  | -0.86 (-4.99, 3.04) | 0.65 |
| Overall survival | Premenopasual | 91/381 | HR^5^ | 1.02 (0.99, 1.05) | 0.29 |
|  | Postmenopasual | 176/539 |  | 0.99 (0.96, 1.01) | 0.31 |
| Breast cancer-specific survival | Premenopasual | 69/381 | SHR^6^ | 1.02 (0.98, 1.05) | 0.33 |
|  | Postmenopasual | 70/539 |  | 1.00 (0.96, 1.05) | 0.99 |

^1^ Effect estimates are presented per 1-year increase in lifetime endogenous hormone exposure (LHEendo).

^2^ Odds ratios (ORs) were estimated from ordinal logistic regression models for TIL level.

^3^ CD4+ and CD8+ T cell densities were treated as continuous variables. Generalized linear models with a Gamma distribution and log link function were used to assess the associations between LHEendo with CD4+ and CD8+ T cell densities among participants with positive density values. Results are reported as percent changes in expected mean density per 1-year increase in endogenous hormone exposure.

^4^ Models adjusted for race, family history of breast cancer and duration of oral contraceptive use. Age at diagnosis was not included in the stratified models. In premenopausal women, LHEendo was defined as age at diagnosis minus age at menarche: adding age at diagnosis results in multicollinearity, with variance inflation factors (VIFs) >10 in the TILs categories, CD4+ T cell density, CD8+ T cell density and survival models. In postmenopausal women, LHEendo was defined as age at menopause minus age at menarche, and the exposure window was completed before diagnosis; therefore, age at diagnosis was not included in the DAG-defined adjustment set.

^5^ Hazard ratios (HRs) were estimated from Cox proportional hazards models for overall survival.

^6^ Subdistribution hazard ratio (SHRs) were estimated from Fine-Gray competing risks models for breast cancer specific survival.

^7^ N for overall survival and Breast cancer-specific survival presented as the number of events/the number of total observations

CI, confidence interval; TILs, tumor-infiltrating lymphocytes

**Supplementary Table S10 Stratified analyses of endogenous hormone exposure in relation to TILs, CD4⁺ T cells, and CD8⁺ T cells by race and molecular subtype**

| **Subgroup** | **N** | **OR or percent change** | | **95% CI** | **P-value** | **P-interaction^5^** |
| --- | --- | --- | --- | --- | --- | --- |
| ***TIL level*^3^** |  |  | |  |  |  |
| **Race^1^** |  |  | |  |  | 0.54 |
| Black | 834 | OR = 0.99 | | (0.97, 1.01) | 0.20 |  |
| White | 335 | OR = 0.98 | | (0.95, 1.01) | 0.12 |  |
| **Molecular subtype^2^** |  |  | |  |  | 0.23 |
| HR+/HER2+ | 142 | OR = 1.02 | | (0.97, 1.06) | 0.46 |  |
| HR+/HER2- | 688 | OR = 0.98 | | (0.96, 1.00) | **0.022** |  |
| HR-/HER2+ | 70 | OR = 1.02 | | (0.96, 1.08) | 0.52 |  |
| HR-/HER2- | 209 | OR = 0.98 | | (0.95, 1.01) | 0.24 |  |
|  |  |  | |  |  |  |
| ***CD4+ T cell density***^4^ |  | |  |  |  |  |
| **Race^1^** |  |  | |  |  | 0.84 |
| Black | 426 | -1.01% | | (-2.72, 0.73) | 0.25 |  |
| White | 107 | -1.41% | | (-4.99, 2.32) | 0.45 |  |
| **Molecular subtype^2^** |  |  | |  |  | 0.65 |
| HR+/HER2+ | 61 | 0.62% | | (-4.07, 5.54) | 0.80 |  |
| HR+/HER2- | 330 | -1.86% | | (-3.89, 0.21) | 0.08 |  |
| HR-/HER2+ | 32 | 0.65% | | (-5.37, 7.06) | 0.84 |  |
| HR-/HER2- | 97 | -0.06% | | (-3.70, 3.71) | 0.97 |  |
|  |  |  | |  |  |  |
| ***CD8+ T cell density***^4^ |  |  | |  |  |  |
| **Race^1^** |  |  | |  |  | 0.54 |
| Black | 446 | -2.44% | | (-4.86, 0.05) | 0.05 |  |
| White | 131 | -4.03% | | (-8.73, 0.92) | 0.11 |  |
| **Molecular subtype^2^** |  |  | |  |  | 0.74 |
| HR+/HER2+ | 68 | -2.65% | | (-8.80, 3.92) | 0.42 |  |
| HR+/HER2- | 347 | -2.12% | | (-4.94, 0.78) | 0.15 |  |
| HR-/HER2+ | 35 | 2.07% | | (-6.25, 11.13) | 0.64 |  |
| HR-/HER2- | 113 | -3.71% | | (-8.11, 0.91) | 0.11 |  |

^1^ Models were adjusted for race (included as an interaction term), family history of breast cancer and the duration of oral contraceptive use.

^2^ Models were adjusted for molecular subtype (included as an interaction term), race, family history of breast cancer and the duration of oral contraceptive use.

^3^ TIL level were modeled using ordinal logistic regression and reported as odds ratio (ORs) for being in a higher TIL category per 1-year increase in endogenous hormone exposure.

^4^ CD4+ and CD8+ T cell densities were treated as continuous variables. Generalized linear models with a Gamma distribution and log link function were used to assess the associations between LHEendo and CD4+ or CD8+ T cell densities among participants with positive density values. Results were reported as percent changes in expected mean density per 1-year increase in endogenous hormone exposure.

^5^ P value of the likelihood ratio χ2 test for interaction.

CI, confidence interval; TIL, Tumor-infiltrating lymphocyte

**Supplementary Table S11 Stratified analyses of** **exogenous hormone exposure in relation to TILs, CD4⁺ T cells, and CD8⁺ T cells by race and molecular subtype**

| **Subgroup** | **N** | **OR or percent change** | | **95% CI** | | | **P-value** | | | **P-interaction^5^** | | |
| --- | --- | --- | --- | --- | --- | --- | --- | --- | --- | --- | --- | --- |
| ***TIL level*^3^** |  |  | |  | | |  | | |  | | |
| **Race^1^** |  |  | |  | | |  | | | 0.07 | | |
| Black | 839 | OR = 1.01 | | (0.99, 1.03) | | | 0.22 | | |  | | |
| White | 337 | OR = 0.98 | | (0.96, 1.01) | | | 0.17 | | |  | | |
| **Molecular subtype^2^** |  |  | |  | | |  | | | 0.29 | | |
| HR+/HER2+ | 145 | OR = 0.96 | | (0.91, 1.01) | | | 0.09 | | |  | | |
| HR+/HER2- | 689 | OR = 1.00 | | (0.98, 1.02) | | | 0.76 | | |  | | |
| HR-/HER2+ | 69 | OR = 1.01 | | (0.96, 1.07) | | | 0.59 | | |  | | |
| HR-/HER2- | 213 | OR = 1.01 | | (0.98, 1.05) | | | 0.45 | | |  | | |
|  |  |  | |  | | |  | | |  | | |
| ***CD4+ T cell density***^4^ |  | |  | | |  | | |  | | |  |
| **Race^1^** |  |  | |  | | |  | | | 0.15 | | |
| Black | 432 | 0.44% | | (-1.27, 2.17) | | | 0.62 | | |  | | |
| White | 109 | -2.18% | | (-5.37, 1.12) | | | 0.19 | | |  | | |
| **Molecular subtype^2^** |  |  | |  | | |  | | | 0.25 | | |
| HR+/HER2+ | 63 | -2.67% | | (-7.44, 2.36) | | | 0.29 | | |  | | |
| HR+/HER2- | 335 | -1.02% | | (-3.01, 1.01) | | | 0.32 | | |  | | |
| HR-/HER2+ | 31 | 3.40% | | (-1.61, 8.68) | | | 0.19 | | |  | | |
| HR-/HER2- | 99 | 0.31% | | (-3.45, 4.21) | | | 0.87 | | |  | | |
|  |  |  | |  | | |  | | |  | | |
| ***CD8+ T cell density***^4^ |  |  | | |  | | |  | | |  | |
| **Race^1^** |  |  | |  | | |  | | | 0.41 | | |
| Black | 452 | 0.91% | | (-1.61, 3.49) | | | 0.48 | | |  | | |
| White | 133 | -1.19% | | (-5.63, 3.45) | | | 0.61 | | |  | | |
| **Molecular subtype^2^** |  |  | |  | | |  | | | 0.36 | | |
| HR+/HER2+ | 70 | -4.02% | | (-10.70, 3.16) | | | 0.26 | | |  | | |
| HR+/HER2- | 352 | 1.20% | | (-1.64, 4.13) | | | 0.41 | | |  | | |
| HR-/HER2+ | 34 | 4.19% | | (-3.07, 11.99) | | | 0.26 | | |  | | |
| HR-/HER2- | 115 | -1.25% | | (-6.25, 4.01) | | | 0.63 | | |  | | |

^1^ Models were adjusted for race (included as an interaction term), age at diagnosis, menopausal status, and family history of breast cancer.

^2^ Models were adjusted for molecular subtype (included as an interaction term), age at diagnosis, menopausal status, race, and family history of breast cancer.

^3^ TIL level were modeled using ordinal logistic regression and reported as odds ratio (ORs) for being in a higher TIL category per 1-year increase in exogenous hormone exposure.

^4^ CD4+ and CD8+ T cell densities were treated as continuous variables. Generalized linear models with a Gamma distribution and log link function were used to assess the associations between LHEexo and CD4+ or CD8+ T cell densities among participants with positive density values. Results were reported as percent changes in expected mean density per 1-year increase in exogenous hormone exposure.

^5^ P value of the likelihood ratio χ2 test for interaction.

CI, confidence interval; TIL, Tumor-infiltrating lymphocyte

**A**

**B**

**C**

**Supplementary Figure S1. Distribution of stromal Tumor-Infiltrating Lymphocyte (TIL) level (A), CD4+ T cell density (B), and CD8+ T cell density (C)**

**Supplementary Figure S2. Directed acyclic graph (DAG) for endogenous hormone exposure and TILs, CD4⁺ T cell density, and CD8⁺ T cell density among premenopausal women.** The DAG illustrates the hypothesized relationships among LHEendo, breast cancer diagnosis, tumor immune markers (TILs, CD4+ T cell density, or CD8+ T cell density), and related clinical and demographic factors among premenopausal women. The red paths from LHEendo to tumor immune markers highlight potential noncausal paths that may arise due to the study population restricting to women diagnosed with breast cancer. Based on this DAG, breast cancer diagnosis acts as a collider because multiple factors, including OC use duration, family history of breast cancer, and unmeasured breast cancer risk factors, may influence the likelihood of breast cancer diagnosis. Before restricting to breast cancer cases, these paths are closed because the arrows point into the breast cancer diagnosis node. However, conditioning on breast cancer diagnosis may open these otherwise closed paths and induce a noncausal association between LHEendo and tumor immune markers. In this DAG, adjustment for family history and OC use duration can help block these noncausal paths between LHEendo and tumor immune markers. Variables filled in red color, including race, family history of breast cancer, and OC use duration, were included in the adjustment set. AJCC stage, tumor grade, and molecular subtype were not included in the adjustment set because they were considered downstream variables that may be influenced by pre-diagnostic hormone exposure. BMI at enrollment was also not adjusted for because it was measured after breast cancer diagnosis and therefore was not considered a confounder. Age at diagnosis was not included in the LHEendo model among premenopausal women because LHEendo was defined in relation to age at diagnosis, and additional adjustment for age introduced multicollinearity. Abbreviations: LHEendo, lifetime endogenous hormone exposure; TILs, tumor-infiltrating lymphocytes; BMI, body mass index; OC, oral contraceptives; HRT, hormone replacement therapy; AJCC, American Joint Committee on Cancer.

**Supplementary Figure S3. Directed acyclic graph (DAG) for endogenous hormone exposure and TILs, CD4⁺ T cell density, and CD8⁺ T cell density among postmenopausal women.** The DAG illustrates the hypothesized relationships among LHEendo, breast cancer diagnosis, tumor immune markers (TILs, CD4+ T cell density, or CD8+ T cell density), and related clinical and demographic factors among postmenopausal women. The red paths from LHEendo to tumor immune markers highlight potential noncausal paths that may arise due to the study population restricting to women diagnosed with breast cancer. Based on this DAG, breast cancer diagnosis acts as a collider because multiple factors, including OC use duration, family history of breast cancer, and unmeasured breast cancer risk factors, may influence the likelihood of breast cancer diagnosis. Before restricting to breast cancer cases, these paths are closed because the arrows point into the breast cancer diagnosis node. However, conditioning on breast cancer diagnosis may open these otherwise closed paths and induce a noncausal association between LHEendo and tumor immune markers. In this DAG, adjustment for family history and OC use duration can help block these noncausal paths between LHEendo and tumor immune markers. Variables filled in red color, including race, family history of breast cancer, and OC use duration, were included in the adjustment set. AJCC stage, tumor grade, and molecular subtype were not included in the adjustment set because they were considered downstream variables that may be influenced by pre-diagnostic hormone exposure. BMI at enrollment was also not adjusted for because it was measured after breast cancer diagnosis and therefore was not considered a confounder. Age at diagnosis was not included in the LHEendo model among postmenopausal women because LHEendo was defined as age at menopause minus age at menarche, and this exposure window was completed before diagnosis. Abbreviations: LHEendo, lifetime endogenous hormone exposure; TILs, tumor-infiltrating lymphocytes; BMI, body mass index; OC, oral contraceptives; HRT, hormone replacement therapy; AJCC, American Joint Committee on Cancer.

**Supplementary Figure S4. Directed acyclic graph (DAG) for exogenous hormone exposure and TILs, CD4⁺ T cell density, and CD8⁺ T cell density.** The DAG illustrates hypothesized relationships among LHEexo, breast cancer diagnosis, tumor immune markers (TILs, CD4+ T cell density, or CD8+ T cell density), and related clinical and demographic factors. The red paths from LHEexo to tumor immune markers highlight potential noncausal paths that may arise due to the study population restricting to women diagnosed with breast cancer. In this DAG, breast cancer diagnosis acts as a collider because multiple factors, including LHEexo, family history of breast cancer, and unmeasured breast cancer risk factors, may influence the likelihood of breast cancer diagnosis. Before restricting to breast cancer cases, these paths are closed because the arrows point into the breast cancer diagnosis node. However, conditioning on breast cancer diagnosis may open these otherwise closed paths and induce a noncausal association between LHEexo and tumor immune markers. Variables filled in red, including race, family history of breast cancer, age at diagnosis, and menopausal status at diagnosis, were included in the adjustment set to address measured noncausal paths between LHEexo and tumor immune markers. AJCC stage, tumor grade, and molecular subtype were not included in the adjustment set because they were considered downstream variables that may be influenced by pre-diagnostic hormone exposure. BMI at enrollment was also not adjusted for because it was measured after breast cancer diagnosis and therefore was not considered a confounder. Abbreviations: LHEexo, lifetime exogenous hormone exposure; TILs, tumor-infiltrating lymphocytes; BMI, body mass index; AJCC, American Joint Committee on Cancer.

**Supplementary Figure S5. Directed acyclic graph (DAG) for endogenous hormone exposure and overall survival among premenopausal women.** The DAG illustrates the hypothesized relationships among LHEendo, breast cancer diagnosis, overall survival, and related clinical and demographic factors among premenopausal women. The red paths from LHEendo to overall survival highlight potential noncausal paths that may arise due to the study population restricting to women diagnosed with breast cancer. Based on this DAG, breast cancer diagnosis acts as a collider because multiple factors, including OC use duration, family history of breast cancer, and unmeasured breast cancer risk factors, may influence the likelihood of breast cancer diagnosis. Before restricting to breast cancer cases, these paths are closed because the arrows point into the breast cancer diagnosis node. However, conditioning on breast cancer diagnosis may open these otherwise closed paths and induce a noncausal association between LHEendo and overall survival. In this DAG, adjustment for family history and OC use duration can help block these noncausal paths between LHEendo and overall survival. Variables filled in red color, including race, family history of breast cancer, and OC use duration, were included in the adjustment set. AJCC stage, tumor grade, molecular subtype, and treatment were not included in the adjustment set because they were considered downstream variables that may be influenced by pre-diagnostic hormone exposure. BMI at enrollment was also not adjusted for because it was measured after breast cancer diagnosis and therefore was not considered a confounder. Age at diagnosis was not included in the LHEendo model among premenopausal women because LHEendo was defined in relation to age at diagnosis, and additional adjustment for age introduced multicollinearity. Abbreviations: LHEendo, lifetime endogenous hormone exposure; BMI, body mass index; OC, oral contraceptives; HRT, hormone replacement therapy; AJCC, American Joint Committee on Cancer.

**Supplementary Figure S6. Directed acyclic graph (DAG) for endogenous hormone exposure and overall survival among postmenopausal women.** The DAG illustrates the hypothesized relationships among LHEendo, breast cancer diagnosis, overall survival, and related clinical and demographic factors among postmenopausal women. The red paths from LHEendo to overall survival highlight potential noncausal paths that may arise due to the study population restricting to women diagnosed with breast cancer. Based on this DAG, breast cancer diagnosis acts as a collider because multiple factors, including OC use duration, family history of breast cancer, and unmeasured breast cancer risk factors, may influence the likelihood of breast cancer diagnosis. Before restricting to breast cancer cases, these paths are closed because the arrows point into the breast cancer diagnosis node. However, conditioning on breast cancer diagnosis may open these otherwise closed paths and induce a noncausal association between LHEendo and overall survival. In this DAG, adjustment for family history and OC use duration can help block these noncausal paths between LHEendo and overall survival. Variables filled in red color, including race, family history of breast cancer, and OC use duration, were included in the adjustment set. AJCC stage, tumor grade, molecular subtype, and treatment were not included in the adjustment set because they were considered downstream variables that may be influenced by pre-diagnostic hormone exposure. BMI at enrollment was also not adjusted for because it was measured after breast cancer diagnosis and therefore was not considered a confounder. Age at diagnosis was not included in the LHEendo model among postmenopausal women because LHEendo was defined as age at menopause minus age at menarche, and this exposure window was completed before diagnosis. Abbreviations: LHEendo, lifetime endogenous hormone exposure; BMI, body mass index; OC, oral contraceptives; HRT, hormone replacement therapy; AJCC, American Joint Committee on Cancer.

**Supplementary Figure S7. Directed acyclic graph (DAG) for exogenous hormone exposure and overall survival.** The DAG illustrates hypothesized relationships among LHEexo, breast cancer diagnosis, overall survival, and related clinical and demographic factors. The red paths from LHEexo to overall survival highlight potential noncausal paths that may arise due to the study population restricting to women diagnosed with breast cancer. In this DAG, breast cancer diagnosis acts as a collider because multiple factors, including LHEexo, family history of breast cancer, and unmeasured breast cancer risk factors (including health awareness and socioeconomical status), may influence the likelihood of breast cancer diagnosis. Before restricting to breast cancer cases, these paths are closed because the arrows point into the breast cancer diagnosis node. However, conditioning on breast cancer diagnosis may open these otherwise closed paths and induce a noncausal association between LHEexo and overall survival. Variables filled in red, including race, family history of breast cancer, age at diagnosis, and menopausal status at diagnosis, were included in the adjustment set to address measured noncausal paths between LHEexo and overall survival. Screening, AJCC stage, tumor grade, molecular subtype, and treatment were not included in the adjustment set because they were considered downstream variables that may be influenced by pre-diagnostic hormone exposure. BMI at enrollment was also not adjusted for because it was measured after breast cancer diagnosis and therefore was not considered a confounder. Abbreviations: LHEexo, lifetime exogenous hormone exposure; BMI, body mass index; AJCC, American Joint Committee on Cancer.

**Supplementary Figure S8. Flow diagram of analytic sample selection for TIL, CD4+ and CD8+ T cell analyses.**

DCIS, ductal carcinoma in situ; H&E, hematoxylin and eosin; TIL, tumor infiltration lymphocytes; TMA, tissue microarray.

**A**

**B**

**Supplementary Figure S9. Residual plots for proportional subdistribution hazards assessment in Fine-Gray models for breast cancer-specific survival.** Panel A shows the plot for LHEendo and Panel B shows the plot for LHEexo. Deaths from non-breast cancer causes were treated as competing events. Visual assessment of residual plots did not suggest a clear violation of the proportional subdistribution hazards assumption for LHEendo and LHEexo. LHEendo, lifetime endogenous hormone exposure; LHEexo, lifetime exogenous hormone exposure.

**Reference**

1.	Yao S, Cheng TD, Elkhanany A, Yan L, Omilian A, Abrams SI, et al. Breast Tumor Microenvironment in Black Women: A Distinct Signature of CD8+ T-Cell Exhaustion. J Natl Cancer Inst. 2021;113(8):1036-43.
